# Circulating sex-steroids and *Staphylococcus aureus* nasal carriage in a general male population

**DOI:** 10.1017/S0950268822000735

**Published:** 2022-04-22

**Authors:** Dina B. Stensen, Lars Småbrekke, Karina Olsen, Guri Grimnes, Christopher Sivert Nielsen, Johanna U. Ericson, Gunnar Skov Simonsen, Bjørg Almås, Anne-Sofie Furberg

**Affiliations:** 1 Department of Community Medicine, Faculty of Health Sciences, UiT The Arctic University of Norway, 9037 Tromsø, Norway; 2 Division of Internal Medicine, University Hospital of North Norway, 9038 Tromsø, Norway; 3 Department of Pharmacy, Faculty of Health Sciences, UiT The Arctic University of Norway, 9037 Tromsø, Norway; 4 Department of Microbiology and Infection Control, Division of Internal Medicine, University Hospital of North Norway, 9038 Tromsø, Norway; 5 Endocrinology Research Group, Department of Clinical Medicine, Faculty of Health Sciences, UiT The Arctic University of Norway, 9038 Tromsø, Norway; 6 Division of Ageing and Health, Norwegian Institute of Public Health, Oslo, Norway; 7 Department of Pain Management and Research, Division of Emergencies and Intensive Care, Oslo University Hospital, Oslo, Norway; 8 Research Group for Host-Microbe Interactions, Department of Medical Biology, Faculty of Health Sciences, UiT The Arctic University of Norway, 9037 Tromsø, Norway; 9 Hormone Laboratory, Department of Medical biochemistry and pharmacology, Haukeland University Hospital, 5009 Bergen, Norway; 10 Faculty of Health Sciences and Social Care, Molde University College, 6410 Molde, Norway

**Keywords:** Circulating sex-steroids, population-based study, *Staphylococcus aureus* carriage, testosterone

## Abstract

Male sex is associated with higher risk of both colonisation and infection with *Staphylococcus aureus* (*S. aureus*). However, the role of sex-steroids in colonisation among men is largely unknown. Thus, the aim of this study was to investigate possible associations between circulating sex-steroids and nasal carriage of *S. aureus* in a general male population. The population-based Tromsø6 study (2007–2008) included 752 males aged 31–87 years with serum sex-steroids measured by liquid chromatography tandem mass spectrometry and two nasal swab samples for the assessment of *S. aureus* carriage. Multivariable logistic regression models were used to study the association between sex-steroid concentrations and *S. aureus* persistent nasal carriage (two positive swabs *vs.* others), while adjusting for potential confounding factors.

*S. aureus* persistent nasal carriage prevalence was 32%. Among men aged 55 years and above (median age 65 years), there was an inverse dose-response relationship between serum concentration of testosterone and persistent nasal carriage, and carriers had significantly lower mean levels of testosterone (*P* = 0.028, OR = 0.94 per nmol/l change in testosterone; 95% CI = 0.90–0.98). This association was attenuated when adjusting for body mass index and age (OR = 0.96 per nmol/l change in testosterone; 95% CI = 0.91–1.01). There was no association in the total population. This large population-based study suggests that testosterone levels may be inversely related to *S. aureus* persistent nasal carriage in older men. Future studies addressing biological mechanisms underlying the male predisposition to *S. aureus* colonisation and infection may foster preventive interventions that take sex-differences into account.

## Background

Epidemiological research has shown that men are at increased risk of several different infectious diseases [1]. However, data addressing the underlying biological mechanisms are scarce. *Staphylococcus aureus* (*S. aureus*) is more frequent in men compared to women, both as a nasal coloniser and as a causative infectious agent [2, 3]. Nasal colonisation is a major risk factor for *S. aureus* infection [4]. Thus, the identification of biological pathways underlying sex differences in nasal colonisation is important not only to enable a better understanding of host factors in colonisation but also to enable the development of preventive interventions that take sex differences into account.

It is well known that immune functions differ by sex and age [5, 6]. Sex-steroids are key regulators of both the innate and adaptive immune system, and hormone levels and actions are context (i.e. sex and age) dependent. Recently, we showed for the first time that higher levels of circulating testosterone in adult women [7] and use of progestin-only contraceptives (structurally related to testosterone) in younger women [8] are associated with lower prevalence of *S. aureus* nasal carriage. To our knowledge, no epidemiological study has examined whether endogenous sex-hormone levels are associated with *S. aureus* nasal carriage among men.

Thus, the aim of this study was to examine possible associations between endogenous sex-steroids and *S. aureus* nasal carriage in a large male population sample.

## Methods

We used data from male participants in the population-based Tromsø6 study (2007–2008), North Norway, 66% attendance. The study included measurement of height and weight, blood samples and interview and questionnaire on lifestyle and health. Trained nurses at the 6th Tromsø Study screening centre collected nasal swab samples from 1741 male participants. Each nasal vestibule was sampled with the same NaCl (0.9%)-moistened sterile rayon-tipped swab which was rotated three times. The swabs were immediately placed in transport medium (Amies Copan, Brescia, Italy) and stored at 4 °C for a maximum of 3 days. Personnel at the Department of Microbiology and Infection Control, University Hospital of North Norway, (UNN) Tromsø analysed the microbiological samples. The specimens were cultured on blood agar (Oxoid, UK) for growth control and chromID-plates (SAID) for *S. aureus* detection (bioMérieux, Marcy I'Etoile, France). The agar plates were incubated for 42–48 h at 37 °C. To retain high specificity, colony morphology was examined, and the most dominating colony type on the SAID plate was confirmed as *S. aureus* by the Staphaurex plus agglutination test (Murex Diagnostic Ltd, Dartford, UK). Growth of any bacterial colonies on agar plates was registered as a valid culture. A second set of nasal swabs was collected with a median interval of 28 days.

Among the 1741 male participants that provided a nasal swab sample, serum concentrations of sex-steroids were measured in 888 individuals (because of limited funding and additional consent for blood sampling). After exclusion of 19 individuals taking antibiotics the last 24 h and 117 individuals with only one nasal sample, 752 men were included in the present analysis.

Liquid chromatography tandem mass spectrometry (LCMS/MS) was used to measure serum concentrations of testosterone, androstenedione, 17*α*-hydroxyprogesterone (17-OH progesterone) and progesterone [7]. Serum concentrations of gonadotropins (luteinising hormone (LH) and follicle-stimulating hormone (FSH), binding proteins (sex-hormone binding globulin (SHBG) and albumin)), dehydroepiandrostenedione sulphate (DHEAS) and 25-hydroxyvitamin D were assessed by immunoassay methods. Estimation of bioavailable testosterone (free and albumin-bound testosterone) was performed using the equation ‘(testosterone/SHBG) × 10’ [9].

Statistical analyses were performed using Stata/MP 15.1 for Macintosh, with significance level set to *P* < 0.05. Univariable associations were assessed by *χ*^2^ test, Student's *t* test, or Mann–Whitney *U* test. Multivariable logistic regression models were fitted to estimate odds ratios (ORs) and 95% confidence intervals (CIs) for *S. aureus* persistent nasal carriage by change in sex-steroid concentrations, while adjusting for potential confounders. A sensitivity analysis on an age-stratified population (cut-off 55 years, median age) was performed, as both concentration of serum androgens and *S. aureus* persistent nasal carriage were inversely related to age. DAGitty 3.0 was used for model selection, and possible interactions were assessed for in the final model.

## Results

Among the 752 males, age 31–87 years, the prevalence of *S. aureus* persistent nasal carriage was 32%. Persistent nasal carriers were younger, had lower vitamin D levels and lower prevalence of current smoking than others (intermittent or non-carriers; results not shown).

We found no association between any circulating sex-steroid and *S. aureus* nasal carriage in the total population when adjusting for age and body mass index (BMI) in a multivariable logistic regression model (Table 1).

**Table 1.** Associations between hormonal status and *S. aureus* persistent nasal carriage in men

	Persistent nasal carriage^a^ OR^b^ (95% CI)
Testosterone, nmol/l	0.98 (0.95–1.01)
Bioavailable testosterone, nmol/l	0.96 (0.83–1.12)
Androstenedione, nmol/l	1.03 (0.91–1.17)
Dehydroepiandrostenedione, nmol/l	0.96 (0.89–1.03)
17*α*-hydroxyprogesterone, nmol/l	0.97 (0.87–1.08)
Progesterone, nmol/l	1.11 (0.62–1.98)
Sex-hormone binding globulin, nmol/l	0.99 (0.98–1.00)
Albumin, nmol/l	0.96 (0.88–1.04)
Luteinising hormone, IU	0.99 (0.94–1.03)
Follicle-stimulating hormone, IU	1.00 (0.98–1.01)

Adjusted odds ratios (OR) and 95% confidence intervals (95% CI) of carriage by one unit increase in serum hormone biomarkers The Tromsø6 study, *n* = 752.

a Persistent nasal carriage: two *S. aureus* culture positive nasal swab samples.

b Adjusted for age and body mass index (BMI) in multivariable logistic regression analysis.

Among men aged 55 and above, persistent nasal carriers had lower mean serum concentration of both testosterone and SHBG compared to others (*P* = 0.028 and 0.052, respectively, Table 2). Men aged 55 and above had lower odds of persistent nasal carriage with lower concentration of testosterone (OR = 0.94 per nmol/l change in testosterone; 95% CI = 0.90–0.98). When adjusting for BMI, the OR for persistent nasal carriage was 0.96 (95% CI = 0.91–1.01) per nmol/l increase in testosterone in the oldest age group (result not shown).

**Table 2.** Serum concentrations of sex-steroids, gonadotropins and binding proteins by *S. aureus* nasal carrier state

	<55 years *n* = 387^a^			≥55 years *n* = 365^a^		
	Persistent carriage *n* = 141	Others^b^ *n* = 246	*P*-value^c^	Persistent carriage *n* = 96	Others^b^ *n* = 269	*P*-value^c^
Testosterone nmol/l	14.73 (5.97)	14.72 (5.63)	0.728	13.22 (4.51)	14.89 (5.90)	0.028
Bioavailable testosterone^d^ nmol/l	4.19 (1.21)	4.18 (1.32)	0.944	2.97 (0.94)	2.94 (0.92)	0.845
Androstenedione nmol/l	3.02 (1.70)	2.89 (1.10)	0.353	2.43 (0.94)	2.50 (1.09)	0.777
Dehydroepiandrostenedione nmol/l	5.51 (2.19)	5.51 (2.62)	0.601	3.29 (2.32)	3.45 (2.10)	0.259
17*α*-hydroxyprogesterone nmol/l	2.56 (1.21)	2.62 (1.59)	0.778	2.49 (1.45)	2.61 (1.79)	0.497
Progesterone nmol/l	0.23 (0.23)	0.22 (0.24)	0.608	0.23 (0.27)	0.23 (0.31)	0.889
Sex-hormone binding globulin nmol/l	37.11 (16.59)	38.22 (16.50)	0.377	47.81 (17.92)	53.25 (21.49)	0.052
Albumin nmol/l	47.69 (2.00)	47.64 (2.42)	0.949	46.17 (2.51)	46.54 (2.36)	0.198
Luteinising hormone IU	4.25 (2.13)	4.87 (3.69)	0.137	7.06 (5.30)	7.18 (5.50)	0.952
Follicle-stimulating hormone IU	5.63 (3.12)	7.05 (10.52)	0.242	13.02 (14.27)	12.59 (13.45)	0.564

Age group (median split) in men. Data are presented as mean (s.d.). The Tromsø6 study.

s.d., standard deviation.

a Number may vary due to missing values.

b Others; Intermittent carriers (one positive nasal samples of two samples in total) or non-carriers (two negative nasal samples of two samples in total).

c Mann–Witney *U* test.

d Calculated by the equation ‘(testosterone/SHBG) × 10’.

**Fig. 1.**
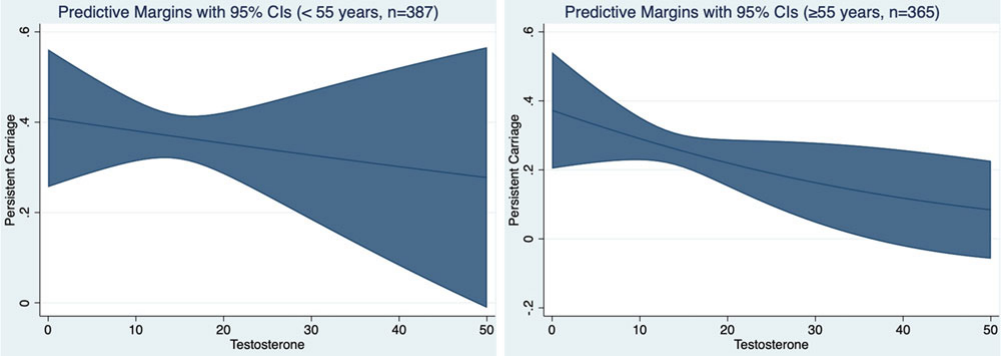
Probability of *S. aureus* persistent nasal carriage according to serum testosterone concentration ((nmol/l), range 0.4–44.3). The Tromsø6 study, male participants.

There was an inverse dose-response relationship between serum testosterone concentration and *S. aureus* persistent carriage. The dose-response relationship was most evident among men aged 55 and above (Fig. 1).

## Discussion

In a recent study among women in the Tromsø6 study, we showed that higher levels of testosterone and bioavailable testosterone were associated with lower prevalence of *S. aureus* nasal carriage [7]. In the present study of the male population, we found no statistically significant associations of sex-steroids, gonadotropins and binding-proteins with the prevalence of *S. aureus* carriage when adjusting for BMI and age. In the age-stratified sensitivity analysis, we found an inverse association for testosterone among the oldest group (≥55 years).

In our population-based data, there was a strong inverse association between age and serum testosterone (results not presented), that is consistent with the described progressive decline in testosterone levels in healthy men between 25 and 75 years [10]. The decline in prevalence of *S. aureus* nasal carriage across adulthood is well known [11]. Both age-related changes in testosterone and bacterial flora may be adaptations to ageing, but the contribution of ageing *per se vs.* lifestyle/nutrition and comorbidities (i.e. confounding factors) to these changes is not clear. Importantly, when adjusting for both age and BMI in our analysis, we found no statistically significant associations between sex-steroid concentrations and *S. aureus* nasal carriage. Thus, we cannot conclude that testosterone is a predictor for *S. aureus* nasal carriage in men.

In this study, we collected only one venous blood sample for analysis of sex-steroid hormones. Male sex-steroid hormones are diurnal, but less so compared to women and this may result in a more representative value with only one measurement. Testosterone in men has a circadian rhythm with optimal sampling from 8 to 10 am. In our study, the blood samples were taken from 8 am to 8 pm, thus attenuating a potential underlying population effect through non-random measurement bias towards the null. Studies have shown that the circadian rhythm is lost in elder men [12], and we believe that the stratified model of men over 55 years of age better represent the true underlying population effect.

We are not able to conclude from our data that circulating sex-steroid concentrations are related to *S. aureus* nasal carriage in men. This is in contrast to our recent findings in women [7], and may represent, among others, imprecision in measurements, a too broad age range, or a different relationship between sex-steroids and immunity in men and women. The role of endogenous sex-steroids in *S. aureus* colonisation should be addressed in future prospective studies. Future studies will benefit on including a larger study size and standardised measurements on sex-steroids.

## Data Availability

The data that support the findings of this study are available from The Tromsø Study but restrictions apply to the availability of these data, which were used under license for the current study, and so are not publicly available. Data are however available from the authors upon request and with permission of The Tromsø Study. Proposals for data should be directed to tromsous@uit.no. Statistical analysis and consent form will be available on request. Proposals should be directed to dina.b.stensen@uit.no.

## References

[1] Washburn TC, Medearis DN Jr. and Childs B (1965) Sex differences in susceptibility to infections. Pediatrics 35, 57–64. 14223226

[2] Olsen K (2012) *Staphylococcus aureus* nasal carriage is associated with serum 25-hydroxyvitamin D levels, gender and smoking status. The Tromso Staph and Skin Study. European Journal of Clinical Microbiology and Infectious Diseases 31, 465–473. 21811869 10.1007/s10096-011-1331-x PMC3303067

[3] Benfield T (2007) Increasing incidence but decreasing in-hospital mortality of adult *Staphylococcus aureus* bacteraemia between 1981 and 2000. Clinical Microbiology and Infection 13, 257–263. 17391379 10.1111/j.1469-0691.2006.01589.x

[4] Bode LG (2010) Preventing surgical-site infections in nasal carriers of *Staphylococcus aureus*. New England Journal of Medicine 362, 9–17. 20054045 10.1056/NEJMoa0808939

[5] Klein SL and Flanagan KL (2016) Sex differences in immune responses. Nature Reviews: Immunology 16, 626–638. 10.1038/nri.2016.90 27546235

[6] Simon AK, Hollander GA and McMichael A (2015) Evolution of the immune system in humans from infancy to old age. Proceedings: Biological Sciences 282, 20143085. 26702035 10.1098/rspb.2014.3085 PMC4707740

[7] Stensen DB (2021) Circulating sex-steroids and *Staphylococcus aureus* nasal carriage in a general female population. European Journal of Endocrinology of the European Federation of Endocrine Societies 184, 337–346. 10.1530/EJE-20-0877 PMC7849480 33428587

[8] Stensen DB (2019) Hormonal contraceptive use and *Staphylococcus aureus* nasal and throat carriage in a Norwegian youth population. PLoS One 14, e0218511. 31276521 10.1371/journal.pone.0218511 PMC6611591

[9] Channer KS and Jones TH (2003) Cardiovascular effects of testosterone: implications of the ‘male menopause’? Heart (British Cardiac Society) 89, 121–122. 12527649 10.1136/heart.89.2.121 PMC1767538

[10] Svartberg J (2003) The associations of age, lifestyle factors and chronic disease with testosterone in men: the Tromso Study. European Journal of Endocrinology of the European Federation of Endocrine Societies 149, 145–152. 10.1530/eje.0.1490145 12887292

[11] Sangvik M (2011) Age- and gender-associated *Staphylococcus aureus* spa types found among nasal carriers in a general population: the Tromso Staph and Skin Study. Journal of Clinical Microbiology 49, 4213–4218. 21998436 10.1128/JCM.05290-11 PMC3232966

[12] Bremner WJ, Vitiello MV and Prinz PN (1983) Loss of circadian rhythmicity in blood testosterone levels with aging in normal men. Journal of Clinical Endocrinology and Metabolism 56, 1278–1281. 6841562 10.1210/jcem-56-6-1278

